# A national, multicenter, retrospective study evaluating retention rate and efficacy of tocilizumab treatment in patients with active rheumatoid arthritis who had an inadequate response to csDMARDs and/or TNF inhibitors

**DOI:** 10.55730/1300-0144.5636

**Published:** 2023-02-18

**Authors:** Güzide Nevsun İNANÇ, Mustafa Ender TERZİOĞLU, Yusuf KARABULUT, Zevcet YILMAZ, Emine Figen TARHAN, Mehmet Emin ENECİK, Ali ŞAHİN, Adem KÜÇÜK, Ayşe AYAN, Metin ÖZGEN, Uğur KARASU, Servet YOLBAŞ

**Affiliations:** 1 Division of Rheumatology, Department of Internal Medicine, Faculty of Medicine, Marmara University, İstanbul, Turkey; 2 Division of Rheumatology, Department of Internal Medicine, Faculty of Medicine, Akdeniz University, Antalya, Turkey; 3 Division of Rheumatology, Department of Internal Medicine, Doruk Yıldırım Hospital, Bursa, Turkey; 4 Division of Rheumatology, Department of Internal Medicine, Tepecik Education and Research Hospital, Health Sciences University, İzmir, Turkey; 5 Division of Rheumatology, Department of Internal Medicine, Faculty of Medicine, Muğla Sıtkı Koçman University, Muğla, Turkey; 6 Division of Rheumatology, Department of Internal Medicine, Medical Park Hospitals, Mersin, Turkey; 7 Division of Rheumatology, Department of Internal Medicine, Faculty of Medicine, Sivas Cumhuriyet University, Sivas, Turkey; 8 Division of Rheumatology, Department of Internal Medicine, Meram Faculty of Medicine, Necmettin Erbakan University, Konya, Turkey; 9 Division of Rheumatology, Department of Internal Medicine, Antalya Training and Research Hospital, Antalya, Turkey; 10 Division of Rheumatology, Department of Internal Medicine, Faculty of Medicine, Ondokuz Mayıs University, Samsun, Turkey; 11 Division of Rheumatology, Department of Internal Medicine, Faculty of Medicine, Pamukkale University, Denizli, Turkey; 12 Division of Rheumatology, Department of Internal Medicine, Faculty of Medicine, İnonü University, Malatya, Turkey

**Keywords:** Rheumatoid arthritis, anti-interleukin-6, tocilizumab

## Abstract

**Background/aim:**

To describe the disease activity and retention rate in rheumatoid arthritis (RA) patients with inadequate response (IR) to conventional synthetic disease-modifying antirheumatic drugs (csDMARDs) and/or tumor necrosis factor inhibitors (TNFis) who were prescribed tocilizumab (TCZ) as first-line or second-line biologic treatment in real-world setting.

**Materials and methods:**

Data gathered from patients’ files was used in a multicenter and retrospective context. Retention rates and the Disease Activity Score in 28 joints with CRP (DAS28-CRP) were evaluated at time points. The relationship of drug efficacy with factors such as smoking, obesity, and previous use of TNFis was also examined.

**Results:**

One hundred and twenty-four patients with a median (IQR) RA duration of 3.7 (7.4) years were included. Mean (SD) age was 52.9 (12.9) and 75% of the patients were female. TCZ retention rates in the 6^th^ and 12^th^ months were 94.1% and 86.6%, respectively. In all patients, DAS28-CRP level decreased significantly from baseline to Months 3 and 6. There was an increase in patients with remission and/or low disease activity and a decrease in patients with high disease activity at Month 3 and Month 6 (p < 0.001 for both). Disease activity was similar between subgroups based on body mass index, smoking status, and previous use of TNFis at any time point. Regression analysis showed that absence of concomitant corticosteroid treatment independently was associated with remission/LDA achievement at Month 6 [OR = 0.31, 95% CI (0.14–0.72), p = 0.006], and Month 12 [OR = 0.35, 95% CI (0.13–0.94), p = 0.037]. Overall, 25 mild adverse events were reported.

**Conclusion:**

TCZ was found to be effective and safe in RA patients with IR to csDMARDs and/or TNFis. The drug retention rate was considered satisfactory with more than half of the patients continuing TCZ treatment at Month 12.

## 1. Introduction

Rheumatoid arthritis (RA) is a chronic inflammatory disease characterized by pain and swelling of peripheral joints resulting in a progressive functional disability and joint damage, as well as an increased risk of osteoporosis and cardiovascular morbidity. The goals of therapy for RA are to decrease joint inflammation and pain, preserve the ability of patients to function in activities of daily living and work, and prevent joint deformity and joint destruction.

Today clinical remission or low disease activity (LDA) is recommended to be aimed by current European Alliance of Associations for Rheumatology (EULAR) and American College of Rheumatology (ACR) guidelines when treating patients with RA [1,2]. Initial treatment with conventional synthetic disease-modifying antirheumatic drugs (csDMARDs), such as methotrexate, has become the standard of care. In the last decades, RA management has dramatically changed with the introduction of biologic DMARD (bDMARD) therapies; tumor necrosis factor inhibitors (TNFis) were the first to be developed [3]. However, approximately 30%–40% of patients develop an inadequate response to csDMARDs and TNFis [4,5]. The increasing knowledge about the pathogenesis of RA and potential targets for treatment resulted in the introduction of new bDMARD options (non-TNF biologics) with different mechanisms of action and targeted synthetic DMARDs (tsDMARDs).

Current guidelines for RA, ACR and the EULAR recommend adding a bDMARD or tsDMARD if the treatment target is not achieved with the first csDMARD strategy. Additionally, if a bDMARD or tsDMARD has failed, treatment with another bDMARD or tsDMARD should be considered. Patients may be given an agent with another mechanism of action or a second TNFi, if there is a failure of TNFi treatment [1,2].

Tocilizumab (TCZ) is a humanized monoclonal antibody against IL-6 receptor which inhibits IL-6 mediated signaling and reduces RA disease symptoms, prevents joint damage, and improves other symptoms such as fatigue, anemia, bone loss, and depression. Besides, it may prevent the development of type 2 diabetes mellitus and increased cardiovascular risk, which are also found to be related to RA [6]. Its efficacy and safety have been demonstrated in many clinical studies, whether in combination with methotrexate [7–9], or in monotherapy [10], and further confirmed with the real-life studies [11–13].

In this retrospective study, we aimed to evaluate the retention rate, efficacy, and safety of TCZ in routine clinical practice in RA patients with inadequate response (IR) to csDMARDs and/or TNFis. The association of drug efficacy in the treatment of TCZ with factors such as smoking, obesity, and previous use of TNFis was also investigated in this study.

## 2. Materials and methods

This study was designed as a national, multicenter, retrospective, and noninterventional study in which data was retrospectively collected between January 2020 and September 2020 with contributions of 13 different rheumatology centers in Turkey. Adult patients with a diagnosis of RA according to the ACR 2010 classification [14] who initiated TCZ between November 30, 2015, and April 02, 2020, and treated with TCZ at least three months after csDMARDs or a TNFi as first or second-line biologic therapy were included in the study. Secondary data were obtained from the patient files and uploaded into an electronic data collection system using electronic data collection forms.

The primary objective was to evaluate the treatment retention rate of TCZ for from 3rd month. Secondary objectives were to analyze the efficacy of TCZ treatment on the 3^rd^, 6^th^, 12^th^, 18^th^, and 24^th^ (±1 month was considered as the time frame for the 3^rd^ month and ±2 months for other time points) months by change in Disease Activity Score in 28 joints with CRP (DAS28-CRP) levels from baseline, to evaluate the efficacy of TCZ treatment in patient subgroups planned according to body mass index (BMI; with a cut-off 30 kg/m^2^), previous use of TNFis, and smoking status. The safety profile of TCZ based on the changes in the lipid profile, liver enzymes, hepatitis B virus (HBV) serology, and latent tuberculosis infection (LTBI) reactivation risk (evaluated with purified protein derivative (PPD) test and/or QuantiFERON-TB Gold Plus test), as well as adverse events over time were also recorded.

Data were obtained for demographics, smoking status (current or nonsmoker), concomitant diseases, disease duration of RA, previous and concomitant RA treatments, laboratory parameters, C-reactive protein (CRP) levels, DAS28-CRP levels, date of TCZ treatment initiation and discontinuation, route of administration, and adverse events. The number of days for TCZ treatment duration was calculated by subtracting the date of starting TCZ treatment from the date of the last visit entered for each patient, and the number of months was calculated by dividing the total number of days by 30. Analysis of total TCZ usage time and retention rate has been made using the last-observation-carried-forward (LOCF) method and as-observed data.

Disease activity per DAS28-CRP score was defined as remission (<2.6), low disease activity (LDA; ≥2.6 and <3.2), moderate disease activity (MDA; ≥3.2 and ≤5.1), and high disease activity (HDA; >5.1). The reference intervals of lipid parameters were used according to the Coronary Heart Disease Protection and Treatment Guideline of the Turkish Society of Cardiology^1^. According to the guideline, total cholesterol [normal (<200 mg/dL), borderline high (200–239 mg/dL), high (≥240 mg/dL)], triglyceride [normal (<150 mg/dL), borderline high (150–199 mg/dL), high (≥200 mg/dL)], LDL-cholesterol [normal (<130 mg/dL), borderline high (130–159 mg/dL), high (160–189 mg/dL), very high (≥190 mg/dL)], and HDL-cholesterol values [normal (≥40 mg/dL for males and ≥50 mg/dL for females)] were categorized for the analysis. The reference intervals for transaminases were based on a study by Ceriotti et al. [15]. The abnormal values of transaminases were graded according to the Common Technology Criteria for Adverse Events (CTCAE) version 4.03^2^: Grade 1, > upper limit of normal (ULN) – 3.0 × ULN; Grade 2, > 3.0 – 5.0 × ULN; Grade 3, > 5.0 – 20.0 × ULN; Grade 4, > 20.0 × ULN. Adverse events were coded using a medical dictionary for regulatory activities (MedDRA) 24.1 for system organ class codes and preferred terms.

This was a secondary data use study, and therefore the treatment retention rate of patients with TCZ treatment for one year was hypothesized as 60% based on literature [16]. Precision was estimated as ±12%, and with a 95% confidence interval, an approximate minimum sample size of 100 patients was required. All data gathered during the course of the study were summarized using descriptive statistics; numeric variables were expressed as mean (standard deviation) or median (minimum and maximum or quartiles); categorical variables were expressed as numbers and percentages. Normality was assessed with visual (histogram and probability graphs) and analytical methods (Kolmogorov-Smirnov/Shapiro-Wilk tests). The relationship between categorical variables was examined with McNemar test. Kaplan-Meier estimates were calculated for the retention rate of TCZ. Logistic regression analysis was carried out to identify the predictors of remission or LDA achievement at months 6 and 12. In this analysis, the explanatory variables used were age, gender, duration of RA, concomitant diseases, the previous number of csDMARDs, previous use of TNFis, concomitant use of csDMARDs, concomitant use of corticosteroids, and being TCZ monotherapy. For statistical analysis, PASW 18.0 for Windows was used. A p-value of <0.05 was considered significant.

## 3. Results

Data of eligible patients who initiated TCZ between November 30, 2015, and April 02, 2020, were collected between January 2020 and September 2020. The study included 124 RA patients (75.0% female) with a mean (SD) age of 52.9 (12.9) at the time of TCZ initiation. The number of patients with follow-up at the 3^rd^, 6^th^, 12^th^, 18^th^, and 24^th^ months were 120 (96.8%), 109 (87.9%), 69 (55.7%), 46 (37.1%), and 24 (19.4%), respectively. The median (IQR) duration of RA was 3.7 (7.4) years. The majority (88.7%) of patients were given TCZ via an intravenous route with a median (Q1–Q3) dose of 600 mg (480 mg–640 mg; n = 109) at baseline. At TCZ initiation, none of the patients were in remission and only four patients (3.2%) were in LDA per DAS28-CRP score. Only 13 patients (10.5%) were given TCZ monotherapy while 111 patients (89.5%) were given TCZ with csDMARDs. During their follow-up, 49 patients (39.5%) used TCZ with glucocorticoids. Patient demographics and baseline disease and treatment characteristics are presented in Table 1.

**Figure 1:**
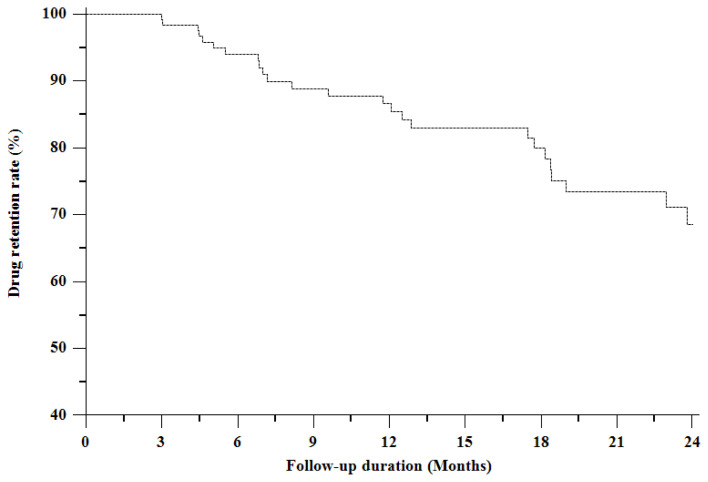
Kaplan–Meier curve of tocilizumab retention rate.

The median duration of TCZ treatment was not reached at 24^th^ months. TCZ retention rates in the 6^th^ and 12^th^ months were 94.1% and 86.6%, respectively (Figure 1). Regression analysis showed that the absence of concomitant corticosteroid treatment independently was associated with remission or LDA achievement at month 6 [OR = 0.31, 95% CI (0.14–0.72), p = 0.006] and month 12 [OR = 0.35, 95% CI (0.13–0.94), p = 0.037] (Table 2).

**Figure 2:**
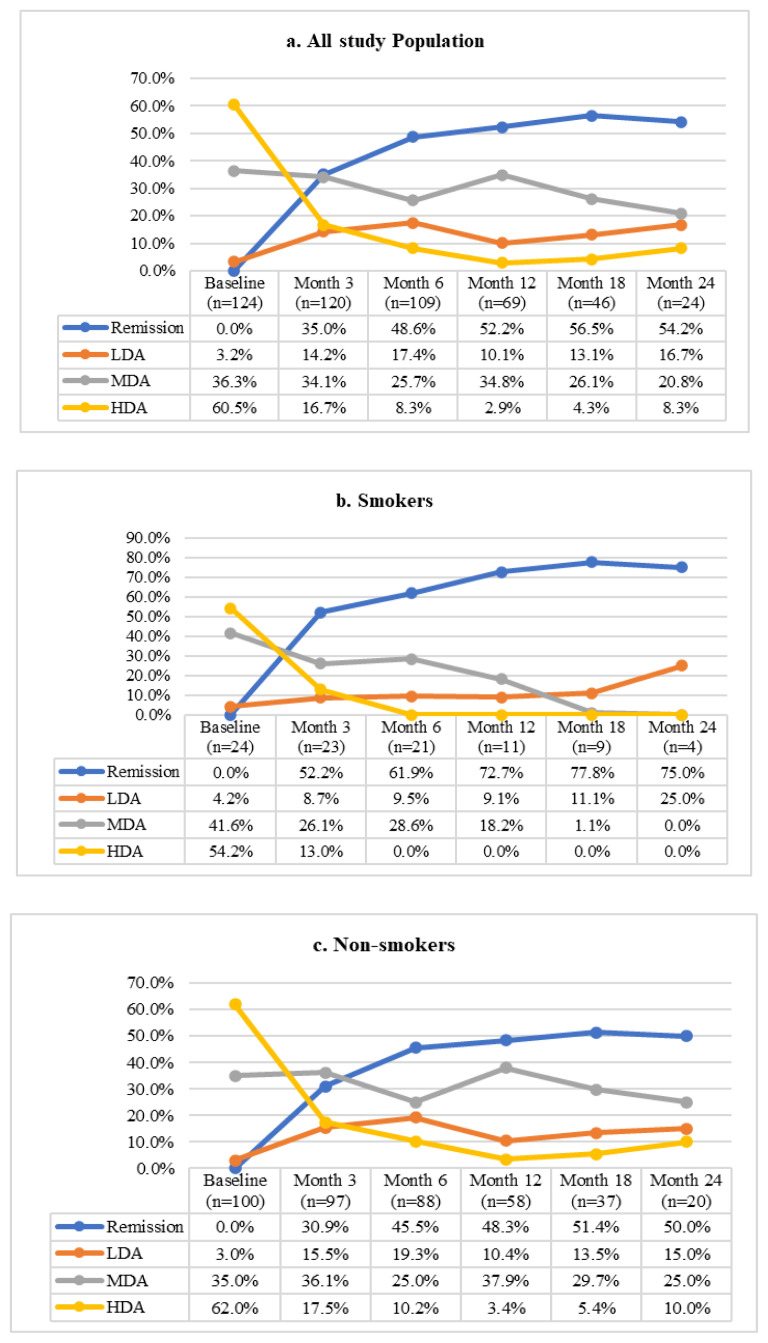

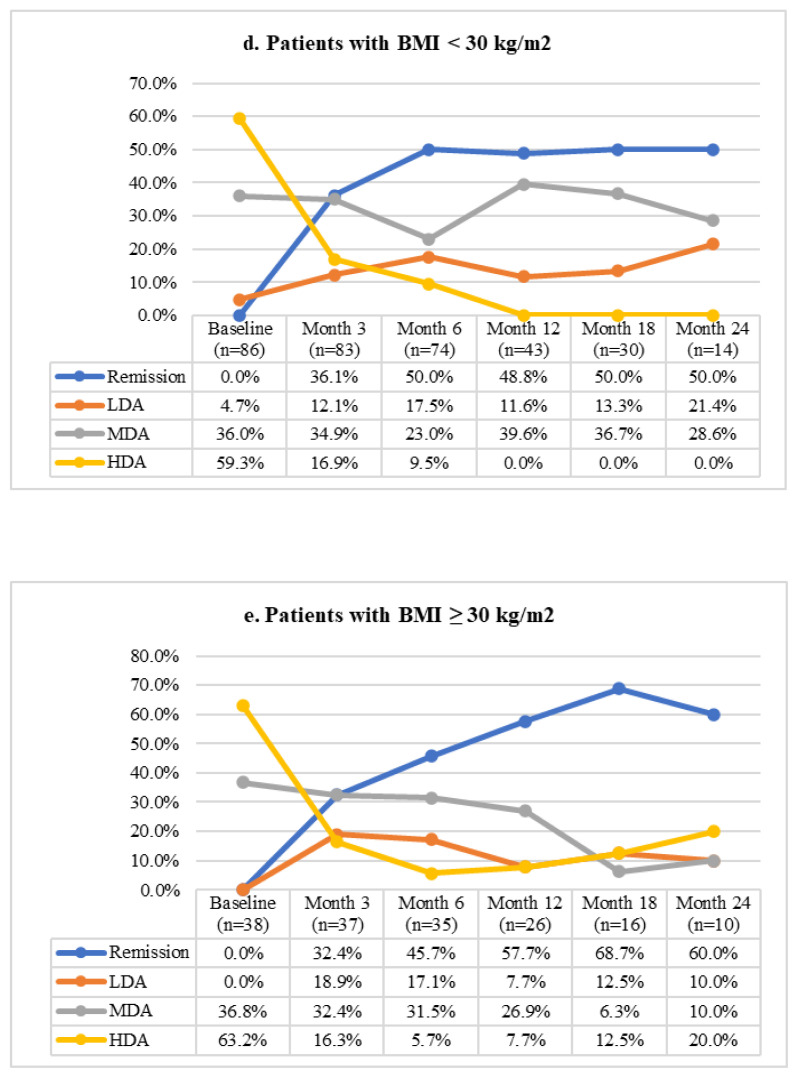
Disease activity per DAS28-CRP over time in (a) all study population, (b) smokers, (c) nonsmokers, (d) patients with BMI < 30 kg/m^2^, (e) patients with BMI ≥ 30 kg/m^2^. **Abbreviations:** HDA: high Disease Activity, LDA: Llow Disease Activity, MDA: Moderate Disease Activity.

The efficacy of TCZ treatment was evaluated by changes in DAS28-CRP levels from baseline at related time points. In all patients, DAS28-CRP level decreased significantly from baseline to months 3 and 6 and there was a significant increase in patients with remission and/or LDA and a decrease in patients with HDA at month 3 and month 6 (p < 0.001 for both, Figure 2a). DAS28-CRP levels were also evaluated at subgroups based on smoking status (smoker and nonsmoker), previous use of TNFis, and BMI (obese: BMI ≥ 30 kg/m^2^ and nonobese: BMI < 30 kg/m^2^). Among the patients, 19.4% were active smokers and 30.6% were obese having a BMI ≥30 kg/m^2^. At baseline, 54.2% of smokers had HDA and 41.6% had MDA, while all of the obese patients had MDA or HDA (36.8% MDA, 63.2% HDA). In both subgroups based on smoking status, patients had significant improvement in disease activity at month 3 (p = 0.013 for smokers and p < 0.001 for nonsmokers) and month 6 (p = 0.041 for smokers and p = 0.005 for nonsmokers) compared to baseline (Figure 2b and 2c). Similar significance was also observed in BMI subgroups: improvement of disease activity at month 3 (p < 0.001 for nonobese patients and p = 0.014 for obese patients) and month 6 (p = 0.001 for nonobese patients) compared to baseline (Figures 2d and 2e). However, disease activity levels did not differ between subgroups based on smoking status and BMI at any time point.

**Figure 3:**
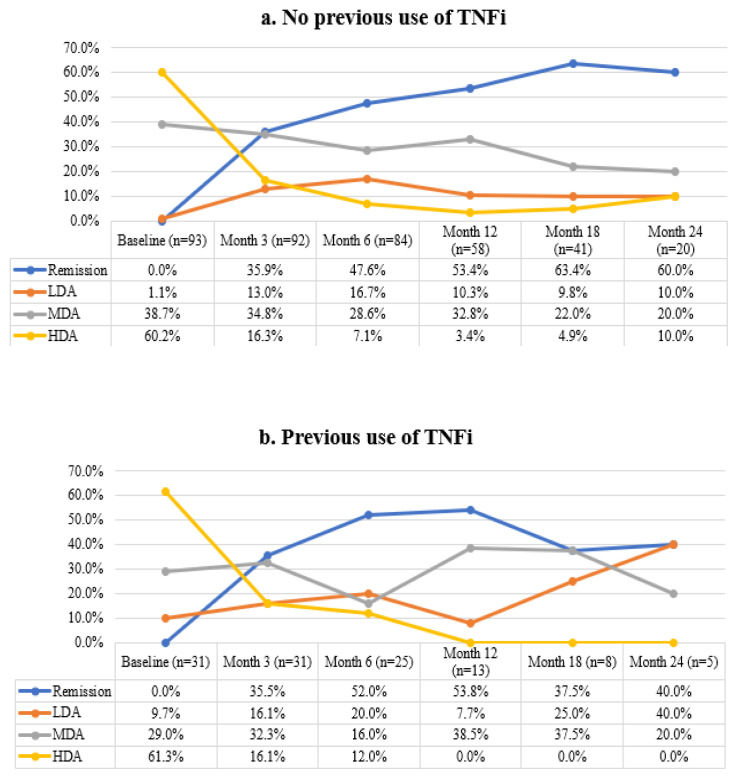
Disease activity per DAS28-CRP over time in (a) patients with no previous use of TNFi, (b) patients with previous use of TNFi. **Abbreviations:** HDA: High Disease Activity, LDA: Low Disease Activity, MDA: Moderate Disease Activity.

At baseline, 31 patients (25%) had previous use of TNFis. Of them, 61.3% had HDA and 29.0% had MDA, which was slightly different than TNFi-naïve subgroup (60.2% HDA and 38.7% MDA, p = 0.051). Although the percentage of patients with remission and/or LDA increased in time compared to baseline, it did not reach a significance level in TNFi-experienced subgroup (p > 0.05 for all time points). However, regarding TNFi-naïve subgroup, there was a significant increase in patients with remission and/or LDA and a decrease in patients with HDA at month 3 (p < 0.001) and month 6 (p = 0.001) compared to baseline (Figures 3a and 3b). In addition, disease activity levels did not differ between subgroups based on TNFi use at related time points (p > 0.05 for all time points).

Lipid profile and liver enzymes were also analyzed during the study. Although the percentage of patients with abnormal total cholesterol and LDL levels increased slightly at month 3, it did not reveal a significant difference (Table 3, p > 0.05 for both). Furthermore, these increments returned to baseline ratios at month 6. The percentages of patients with normal levels of triglycerides and HDL were also similar at related time points compared to baseline (p > 0.05 for all). Similar trends of increment of transaminase levels at month 3 and decrement at month 6 were observed as well (Table 4). Grade ≥ 1 alanine aminotransferase (ALT) was observed in 5.7% and 17.2% of patients at baseline and month 3, respectively (p = 0.004). All other comparisons regarding Grade ≥ 1 ALT and AST (aspartate aminotransferase) were similar at related time points compared to baseline (p > 0.05 for all).

In total, 25 adverse events were reported during the study (Table 5). TCZ treatment was temporarily discontinued in three patients (one for skin rash during TCZ infusion which resolved quickly and two for planned surgeries). There was one death due to the prior malignancy, which was unrelated to the study drug. All other adverse events were mild and resolved completely. The Hepatitis B profile and LTBI reactivation were also evaluated for safety profile. Overall, no hepatitis B infection and LTBI reactivation were recorded during the follow-up.

## 4. Discussion

In this real-world data study, TCZ was found to be effective and safe in RA patients with inadequate response to csDMARDs and/or TNFi treatments. The drug retention rate for TCZ was also considered satisfactory with more than half of the patients continuing TCZ treatment at Month 12.

In our study, approximately nine out of ten patients were still on TCZ treatment at the 6^th^ month, maintaining a rate of 86% at 12 months. Flipo et al. found the median retention rate of TCZ treatment until one year was 69%, without any difference between TCZ monotherapy or combination with csDMARDs [12]. Haraoui et al. recently published a real-world study including 1912 patients from 16 countries, in which 78.7% of the patients were found to continue to receive TCZ treatment [17]. In another study, the drug continuation rate of TCZ was found 89.4% at one year, which was concordant with our study [18].

There are several studies demonstrating the efficacy and safety of TCZ. These studies showed the superiority of TCZ compared to placebo [9–13,19], and to TNFis [20] by disease activity measurements. Similar results were observed in a German cohort including patients refractory to csDMARDs or TNFis, and TCZ in combination with csDMARDs or as monotherapy resulted in significantly more patients achieving remission compared with TNFis [21]. In a recent meta-analysis evaluating the efficacy of targeted immune modulators (Janus kinase inhibitors, TNFis, and other non-TNFi therapies) in patients with IR to csDMARDs, it was shown that TCZ had the highest effect to achieve DAS28 remission among all targeted immune modulators compared with a csDMARD alone [22]. Bykerk et al. found a 56.8% achievement of DAS28 remission with TCZ treatment at 24 weeks in a patient cohort with IR to DMARDs and/or TNFis [23]. In a recent study from the Italian biologics’ register GISEA, remission was achieved in 51% and 52.3% of the patients using TCZ at 6 and 24 months, respectively [24]. Similarly, in our study, approximately 60% of the patients achieved clinical remission or LDA at month 3, and this finding was sustained for two years.

Obesity and smoking are among the important risk factors for RA [25]. Although poor responses were associated with smoking and increased BMI [26,27], recent studies showed that TCZ response was not affected by smoking status or BMI of the patient [28–30]. In our study, improvements in disease activity were similarly observed in patient subgroups based on these factors. Furthermore, the efficacy of the TCZ treatment in TNFi-experienced subgroup was comparable to that in the TNFi-naïve subgroup. Bykerk et al. showed improvements in DAS28 scores with TCZ treatment in the treated population while better outcomes were observed in TNFi-naive patients than TNFi-exposed patients [23]. As a result, both TNFi-naïve and TNFi-experienced patients may benefit from TCZ treatment. Overall, our efficacy results are in line with observations from other studies. Both changes in lipid profile and liver enzymes were acceptable. The treatment with TCZ was well tolerated and overall, the adverse events were as expected from the published records.

Our study has a few limitations. The number of patients refractory to TNFis and at the subgroups were limited. Also, the number of patients on follow-up after 12 months was considerably low as a result of follow-up challenges in routine clinical practice. Additionally, the lack of a comparison group was considered as a limitation of this study. Since this was a retrospective study, data from all patients were not available for several variables.

In conclusion, TCZ drug retention rates of 94.1% and 86.6% at months 6 and 12 respectively were concordant with previously conducted TCZ clinical studies and TCZ seemed to be effectively showing favorable DAS28-CRP response rates in routine clinical practice in patients with moderate to severe RA responding inadequately to csDMARDs and/or TNFis.

## Figures and Tables

**Table 1:** Patient demographics and baseline disease and treatment characteristics at tocilizumab initiation.

Characteristics	
**Age (years), mean (SD)**	52.9 (12.9)
**Female, n (%)**	93 (75.0)
**Duration of RA (years), median (IQR)**	3.7 (7.4)
**BMI (kg/m** **^2^** **), mean (SD)**	27.6 (5.1)
**Current smoker, n (%)**	24 (19.4)
**Concomitant Diseases, n (%)**	
Hypertension	30 (24.2)
Diabetes mellitus	13 (10.5)
Dyslipidemia	5 (4.0)
Coronary artery disease	4 (3.2)
Asthma	3 (2.4)
Chronic obstructive pulmonary disease	1 (0.8)
Chronic renal failure	1 (0.8)
Raynaud disease	1 (0.8)
Benign prostatic hyperplasia	1 (0.8)
**CRP (mg/dL), median (IQR)** a	2.8 (6.4)
**Disease activity per DAS28-CRP score, n (%)**	
Remission	0 (0)
Low disease activity	4 (3.2)
Moderate disease activity	45 (36.3)
High disease activity	75 (60.5)
**Route of TCZ administration, n (%)**	
Intravenous	110 (88.7)
Subcutaneous	14 (11.3)
**Inadequate response, n (%)**	
To csDMARDS	96 (77.4)
To TNFi	28 (22.6)

a For CRP, n = 110.

BMI, Body Mass Index; CRP, C-Reactive Protein; csDMARD, Conventional Synthetic Disease-Modifying Antirheumatic Drug; DAS28-CRP, Disease Activity Score in 28 joints-C-Reactive Protein; IQR, Interquartile Range; RA, Rheumatoid Arthritis; SD, Standard Deviation; TCZ, Tocilizumab; TNFi, Tumor Necrosis Factor Inhibitor.

**Table 2:** Logistic regression analysis of factors affecting remission or low disease activity achievement at month 6 and month 12.

Independent variables	Month 6		Month 12	
	OR (95% Cl)	p-value	OR (95% Cl)	p-value
**Age**	1.00 (0.97–1.03)	0.906	0.97 (0.94–1.01)	0.198
**Gender**				
Male	Reference	-	Reference	-
Female	0.97 (0.37–2.52)	0.943	0.26 (0.05–1.27)	0.095
**Duration of RA**	0.99 (0.97–1.02)	0.654	0.97 (0.94–1.01)	0.137
**Hypertension**	2.24 (0.92–5.48)	0.076	2.50 (0.85–7.33)	0.095
**Diabetes Mellitus**	1.80 (0.56–5.80)	0.327	1.87 (0.42–8.18)	0.410
**Previous use of TNFi**	1.43 (0.54–3.81)	0.476	0.91 (0.26–3.14)	0.879
**Concomitant use of csDMARDs**	0.62 (0.16–2.44)	0.491	1.34 (0.27–6.50)	0.719
**Concomitant use of corticosteroids**	**0.31 (0.14**–**0.72)**	**0.006**	**0.35 (0.13**–**0.94)**	**0.037**
**Being TCZ monotherapy**	1.62 (0.41–6.38)	0.491	0.75 (0.15–3.64)	0.719

CI, Confidence Interval; csDMARD, Conventional Synthetic Disease-Modifying Antirheumatic Drug; OR, Odds Ratio; RA, Rheumatoid Arthritis; TCZ, Tocilizumab; TNFi, Tumor Necrosis Factor Inhibitor

**Table 3:** Lipid profile during the study.

Lipid Profile, n (%)						
Intervals	Baseline	Month 3	Month 6	Month 12	Month 18	Month 24
**Total Cholesterol**						
**N**	26	26	19	22	6	8
**Normal**	15 (57.7)	10 (38.5)	12 (63.1)	9 (40.9)	1 (16.7)	1 (12.5)
**Borderline High**	10 (38.5)	12 (46.1)	4 (21.1)	8 (36.4)	1 (16.7)	6 (75.0)
**High**	1 (3.8)	4 (15.4)	3 (15.8)	5 (22.7)	4 (66.7)	1 (12.5)
**Triglyceride**						
**N**	27	26	19	21	6	8
**Normal**	19 (70.4)	19 (73.1)	11 (57.9)	11 (52.4)	5 (83.3)	5 (62.5)
**Borderline High**	7 (25.9)	4 (15.4)	6 (31.6)	5 (23.8)	-	1 (12.5)
**High**	1 (3.7)	3 (11.5)	2 (10.5)	5 (23.8)	1 (16.7)	2 (25.0)
**Very High**	-	-	-	-	-	-
**LDL Cholesterol**						
**N**	26	22	18	19	4	6
**Normal**	20 (76.9)	15 (68.2)	14 (77.8)	8 (42.1)	1 (25.0)	3 (50.0)
**Borderline High**	4 (15.4)	4 (18.2)	3 (16.7)	6 (31.6)	1 (25.0)	3 (50.0)
**High**	2 (7.7)	2 (9.1)	-	4 (21.1)	-	-
**Very High**	-	1 (4.5)	1 (5.6)	1 (5.3)	2 (50.0)	-
**HDL Cholesterol**						
**N**	26	27	19	22	6	7
**Normal**	13 (50.0)	17 (63.0)	15 (78.9)	16 (72.7)	6 (100.0)	6 (85.7)
**Abnormal**	13 (50.0)	10 (37.0)	4 (21.1)	6 (27.3)	-	1 (14.3)

HDL, High-density Lipoprotein; LDL, Low-density Lipoprotein

**Table 4:** Transaminases of the patients during the study

Transaminases, n (%)						
Intervals	Baseline	Month 3	Month 6	Month 12	Month 18	Month 24
**ALT**						
**N**	105	99	91	63	39	20
**Normal**	99 (94.3)	82 (82.8)	81 (89.0)	59 (93.7)	37 (94.9)	19 (95.0)
**Grade 1**	6 (5.7)	16 (16.2)	9 (9.9)	4 (6.3)	2 (5.1)	1 (5.0)
**Grade 2**	-	1 (1.0)	1 (1.1)	-	-	
**Grade 3**	-	-	-	-	-	
**Grade 4**	-	-	-	-	-	
**AST**						
**N**	91	80	76	54	35	19
**Normal**	87 (95.6)	71 (88.8)	70 (92.1)	51 (94.4)	33 (94.3)	19 (100.0)
**Grade 1**	4 (4.4)	8 (10.0)	6 (7.9)	3 (5.6)	2 (5.7)	-
**Grade 2**	-	-	-	-	-	-
**Grade 3**	-	1 (1.3)	-	-	-	-
**Grade 4**	-	-	-	-	-	-

ALT, Alanine Aminotransferase; AST, Aspartate Aminotransferase

**Table 5:** Adverse events reported in patients during the follow-up.

Adverse events referred to the system organ classes*	Adverse events by preferred term*	N (%)
Musculoskeletal and connective tissue disorders	Musculoskeletal pain	5 (20)
Surgical and medical procedures	Surgery	3 (12)
Musculoskeletal and connective tissue disorders	Joint range of motion decreased	2 (8)
Gastrointestinal disorders	Dry mouth	2 (8)
Infections and infestations	Upper respiratory tract infection	2 (10)
Skin and subcutaneous tissue disorders	Rash	2 (8)
Infections and infestations	Infection	1 (4)
Skin and subcutaneous tissue disorders	Pruritus	1 (4)
Eye disorders	Xerophthalmia	1 (4)
Neoplasms benign, malignant, and unspecified (incl cysts and polyps)	Breast cancer	1 (4)
Psychiatric disorders	Depression	1 (4)
Nervous system disorders	Dizziness	1 (4)
Respiratory, thoracic, and mediastinal disorders	Asthma	1 (4)
Respiratory, thoracic, and mediastinal disorders	Systemic sclerosis pulmonary	1 (4)
Investigations	Mycobacterium tuberculosis complex test positive	1 (4)
**Total**		25 (100)

* For system organ classes codes and preferred terms MedDRA 24.1 was used.

## Footnotes

1 Turkish Society of Cardiology (2002). Coronary Heart Disease Protection and Treatment Guideline [online]. Website https://tkd.org.tr/kilavuz/k11/8_tbl1.htm [accessed 15 November 2022]

2 U.S. Department of Health and Human Services, National Institutes of Health, National Cancer Institute (2009). Common Terminology Criteria for Adverse Events v4.0 (CTCAE) [online]. Website https://evs.nci.nih.gov/ftp1/CTCAE/CTCAE_4.03/CTCAE_4.03_2010-06-14_QuickReference_5x7.pdf [accessed 15 November 2022]
